# Genomics and genetics of gonadotropin beta-subunit genes: Unique *FSHB* and duplicated *LHB*/*CGB* loci

**DOI:** 10.1016/j.mce.2010.04.024

**Published:** 2010-11-25

**Authors:** Liina Nagirnaja, Kristiina Rull, Liis Uusküla, Pille Hallast, Marina Grigorova, Maris Laan

**Affiliations:** a Institute of Molecular and Cell Biology, University of Tartu, Riia St. 23, 51010 Tartu, Estonia; b Department of Obstetrics and Gynecology, University of Tartu, Puusepa 8 G2, 51014 Tartu, Estonia; c Estonian Biocentre, Riia St. 23b, 51010 Tartu, Estonia

**Keywords:** Gonadotropin hormones, *FSHB*, *LHB*, *HCG beta*, Gene duplications, Gene conversion, Selection, Mutations, Polymorphisms, Gene expression

## Abstract

The follicle stimulating hormone (FSH), luteinizing hormone (LH) and chorionic gonadotropin (HCG) play a critical role in human reproduction. Despite the common evolutionary ancestry and functional relatedness of the gonadotropin hormone beta (*GtHB*) genes, the single-copy *FSHB* (at 11p13) and the multi-copy *LHB*/*CGB* genes (at 19q13.32) exhibit locus-specific differences regarding their genomic context, evolution, genetic variation and expressional profile. *FSHB* represents a conservative vertebrate gene with a unique function and it is located in a structurally stable gene-poor region. In contrast, the primate-specific *LHB*/*CGB* gene cluster is located in a gene-rich genomic context and demonstrates an example of evolutionary young and unstable genomic region. The gene cluster is shaped by a constant balance between selection that acts on specific functions of the loci and frequent gene conversion events among duplicons. As the transcription of the *GtHB* genes is rate-limiting in the assembly of respective hormones, the genomic and genetic context of the *FSHB* and the *LHB*/*CGB* genes largely affects the profile of the hormone production.

## 1 Introduction

The follicle stimulating hormone (FSH), luteinizing hormone (LH) and chorionic gonadotropin (CG; HCG in human) are functionally and evolutionarily related hormones regulating reproductive function (Pierce and Parsons, 1981). FSH and LH are produced in the anterior lobe of pituitary gland in a pulsatile manner and their function in gonads is mediated over distinct FSH and LH receptors, respectively (Table 1) (Dalkin et al., 2001; Ascoli et al., 2002; Dias et al., 2002). FSH is required for follicle maturation and stimulation of ovarian estrogen production in women. In males, FSH promotes Sertoli cell proliferation and indirectly spermatogenesis (McGee and Hsueh, 2000; Plant and Marshall, 2001). LH stimulates female progesterone synthesis and ovulation and also theca cell androgen production. In males, LH stimulates testosterone production in Leydig cells (Moyle and Campbell, 1996). Apart from FSH and LH, the evolutionarily young primate-specific CG is synthesised in placenta (Table 1). Although CG and LH bind to the same receptor LH/CGR, their properties are different as CG is more stable (hormone half-life for LH <1 h compared to ∼24 h for HCG), the production of hormone is continuous and pregnancy-specific (Wehmann and Nisula, 1981; Moyle and Campbell, 1996). The main function of CG is to delay apoptosis of the *corpus luteum gravidium*, prepare endometrium for the implantation of the fetus in early pregnancy and promote fetal testicular testosterone production.

FSH, LH and CG are all hetero-dimeric proteins that share a common alpha-subunit, but have a hormone-specific beta-subunit that mediates binding to the respective receptors (Morgan et al., 1975; Moyle et al., 1975; Rathnam and Saxena, 1975). The unique *CGA* gene coding for the alpha-subunit (116 aa) is located at chromosome 6q12–q21. It has a conservative nature in forming a functional hormone with all beta-subunits of gonadotropins as well as thyroid-stimulating hormone, and also contributes to the binding of the three receptors (FSHR, LHR, TSHR) (Pierce et al., 1971). So far no human patients have been described carrying *CGA* variants, although four non-synonymous changes have been predicted to exon 2 (dbSNP, build 131; http://www.ncbi.nlm.nih.gov/projects/SNP/). The genes coding for FSH, LH and CG beta-subunits have a common evolutionary ancestry and belong to gonadotropin hormone beta-subunit gene family (*GtHB*). Loci for FSH beta (denoted *FSHB* in primates, *Fshb* in mammals and *GTH-I* in fishes) and LH beta (denoted *LHB* in primates, *Lhb* in mammals and *GTH-II* in fishes) are conserved among vertebrates and functional genes have been cloned in fishes, amphibians, reptiles, birds and mammals (Gharib et al., 1989; Guzman et al., 1991; Kumar et al., 1995; Rosenfeld et al., 2001; Shen and Yu, 2002; Kawasaki et al., 2003; Watanabe et al., 2007). The beta-subunit of CG (*CGB*) probably emerged from a duplicate *LHB* gene copy approximately 55–35 million years ago before the divergence of New World and Old World monkeys (Talmadge et al., 1984; Policastro et al., 1986; Bailey et al., 1991; Maston and Ruvolo, 2002; reviewed in Henke and Gromoll, 2008; Hallast and Laan, 2009). Only a few sequence changes led to the novel function and different expression pattern of the derived *CGB* gene compared to the ancestral *LHB* (Fiddes and Goodman, 1980; Talmadge et al., 1984). In Old World monkey and great ape lineages additional gene duplication events have occurred within the *LHB*/*CGB* gene cluster, further diversifying this genomic region among species (Maston and Ruvolo, 2002; Fortna et al., 2004; Dumas et al., 2007).

## 2 Comparative genomic context of gonadotropin beta genes

The evolution of single-copy *FSHB* and multi-copy *LHB*/*CGB* loci represent contrasting scenarios of functionally related *GtHB* gene family members, which are physically separated in the genome. The evolution, divergence and diversity patterns of the *GtHB* genes have been determined by the sequence properties and dynamics of the respective genomic regions.

### 2.1 *FSHB* genomic region

**Fig. 1:**
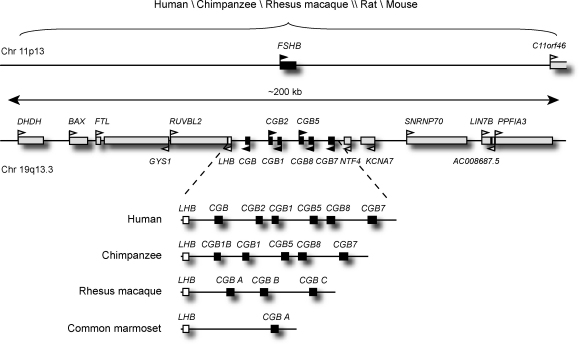
Schematic representation of the genomic context of the *FSHB* and the *LHB*/*CGB* genes (±100 kb). The figure was drawn based on Ensembl database (http://www.ensembl.org/; Release 54). Boxes denote the genes and triangles above or below them point to the direction of transcription. The black boxes and arrowheads indicate *CGB*, white *LHB* and grey neighbouring genes. The *CGB* genes of rhesus macaque (*Macaca mulatta*) and common marmoset (*Callithrix jacchus*) are indicated as *CGB A*–*C*, since their ancestral status relative to the human *CGB* genes is unknown (reviewed in Henke and Gromoll, 2008; Hallast and Laan, 2009).

The beta-subunit of the human FSH (111 aa mature protein) is coded by the *FSHB* gene (4262 bp) at chromosome 11p13. *FSHB* is a single-copy gene located in a G/C-nucleotide deficient and gene-poor genomic region. The G/C content in *FSHB* exonic regions is 43–52% and 30–33% in introns. Only one more gene (*C11org46*; ∼100 kb downstream from *FSHB*) has been mapped within the flanking ±100 kb region (Fig. 1). The closest gene upstream of *FSHB*, *KCNA4*, is located ∼200 kb away. Interestingly, another member of the same gene family, *KCNA7*, is located 11.7 kb downstream of the *LHB*/*CGB* gene cluster at chromosome 19 (Fig. 1). This is consistent with earlier studies showing the common origin of the gonadotropin beta genes (Li and Ford, 1998; Querat et al., 2000; Querat et al., 2001; Querat et al., 2004). The repeat-content of the flanking region of the human *FSHB* gene (∼44%) falls within the average range estimated for the human genome (40–50%) (Lander et al., 2001). The *FSHB* gene and its immediate flanking regions (±100 kb) are characterized by low genome dynamics and high structural conservation since it has preserved very similar features not only in primates but also among mammals (Fig. 1).

### 2.2 *LHB*/*CGB* genomic region

#### 2.2.1 *LHB*/*CGB* genes in human

The human genes coding for the beta-subunits of LH (121 aa mature protein) and HCG (145 aa mature protein) are located in tandem in a shared genomic region (45,165 bp) at 19q13.32 (Fig. 1; Table 1) (Policastro et al., 1986). The *LHB*/*CGB* gene cluster consists of one *LHB* gene (1111 bp), four HCG beta coding genes (*CGB*, *CGB5*, *GGB8* and *CGB7*; 1467 bp) and two gene copies with unknown function (*CGB1* and *CGB2*; 1366 bp) (Policastro et al., 1983, 1986; Fiddes and Talmadge, 1984). In contrast to the gene-poor genomic context of the *FSHB* locus, the *LHB*/*CGB* region is flanked by several genes. In total, 18 annotated genes are mapped within the ±100 kb proximate region of this gene cluster (Fig. 1). The *LHB*/*CGB* region has high G/C-nucleotide content (57% compared to 41% for the human genome average) and is rich in *Alu*-repeats (23.2% compared to 13.1%) (Lander et al., 2001; Hallast et al., 2005, 2008). *Alu*-repeats represent primate-specific repeat elements, which have extensively spread by ‘copy-paste’ mechanism during the past 35–40 million years. These elements may trigger various genomic rearrangements and have been associated with recent abundant duplication events in primate genomes (Bailey et al., 2003).

The structure and sequence content of the human *LHB*/*CGB* gene cluster represents a typical young genomic region evolved by duplication events. There is a high DNA identity between the genes (85–99%) as well as between the inter-genic regions (81–97%) (Hallast et al., 2005). The amino acid sequence identity between the HCG beta-subunit is 98–100% and to LH beta 85% (Bo and Boime, 1992; Hollenberg et al., 1994). The DNA sequence identity between the HCG beta-subunit non-coding genes *CGB1* and *CGB2* and the four HCG beta genes is 85% (Supplementary Figure S1). However, the 132 aa protein predicted for *CGB1* and *CGB2* has no similarity to HCG beta-subunit or to any other known protein due to an alternative open reading frame (Supplementary Figure S2) (Bo and Boime, 1992; Hollenberg et al., 1994; Dirnhofer et al., 1996). Additionally, *CGB1* and *CGB2* have been shown to undergo extensive alternative splicing that potentially encode distinct protein isoforms (Rull et al., 2008a,b; Bo and Boime, 1992).

#### 2.2.2 *LHB*/*CGB* genes in primates

Functionally divergent *CGB* probably emerged from a duplicate *LHB* gene copy in the common ancestor of anthropoid primates 55–35 million years ago and it demonstrates an excellent example of the power of evolution by gene duplication (Ohno, 1970). After the initial gene duplication event, the evolution of the *LHB*/*CGB* gene cluster in New World monkeys (*Platyrrhini*) compared to Old World monkeys, apes and humans (*Catarrhini*) have followed different scenarios (reviewed in detail in Henke and Gromoll, 2008; Hallast and Laan, 2009). All the New World monkey species studied so far (e.g. common marmoset *Callithrix jacchus*) harbour one pseudogenized *LHB* gene and one *CGB* gene coding for dual functions of LH beta and CG beta (Fig. 1) (Simula et al., 1995; Maston and Ruvolo, 2002; Scammell et al., 2008). In Old World monkeys and apes additional duplication events have lead to variable numbers of gene copies among the species. The number of mapped *CGB* genes ranges from three in rhesus macaque (*Macaca mulatta*) to six in humans and potentially even up to ∼50 copies in gorilla (*Gorilla gorilla*) (Crawford et al., 1986; Maston and Ruvolo, 2002; Fortna et al., 2004; Dumas et al., 2007).

The active ongoing genome dynamics in the primate *LHB*/*CGB* locus was further proved by the finding that the *CGB1-like* gene copies are specific only to African great apes (Hallast et al., 2007). Most intriguingly, the upstream region distinct to *CGB1-like* genes harbours a novel small gene *snaR* (small NF90-associated RNA) transcribed from an antisense strand predominantly in testis and weakly in placenta and brain (Parrott and Mathews, 2007). As *CGB1-like* genes, *snaR* orthologs are only present in hominoids, and were shown to descend from *Alu*-transcripts (Parrott and Mathews, 2009). The *CGB1*-specific insert disrupting the ORF of HCG beta originates from a cluster of *snaR* genes mapped to chromosomes 19 in human.

Additionally, it was determined that even human and chimpanzee harbour a discordant number of *CGB* genes (six and five copies, respectively) (Fig. 1) (Hallast et al., 2008). The structural differences of the *LHB*/*CGB* region in two sister-species were best explained by the scenario of independent lineage-specific duplication events.

## 3 Forces affecting diversity and evolution of gonadotropin beta genes

Intra- and interspecies diversification of a genomic segment is influenced by various forces such as selective pressures, mutation rate and DNA sequence rearrangements, but also by demographic history of the species (is not addressed in this review).

### 3.1 Diversity patterns of gonadotropin beta genes

**Fig. 2:**
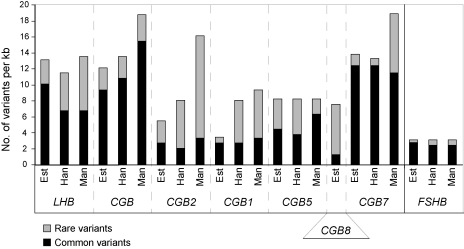
Density of polymorphisms in the human *FSHB* and *LHB*/*CGB* genes. The graph was drawn based on the resequencing data obtained from Hallast et al. (2005) and Grigorova et al. (2007). Bars represent the number of DNA variants calculated per 1000 bp of the re-sequenced region. The length of the analysed region for *LHB*, *CGB*, *CGB2* and *CGB1* genes is 1.5 kb, *CGB5* and *CGB8* 1.6 kb, *CGB7* 2.2 kb and for *FSHB* 2.9 kb. The density fraction of common variants (minor allele frequency, MAF >10%) and rare variants (MAF <10%) is shown by the height of black and grey bars, respectively. Each gene, except for *CGB8*, was analysed in three populations: Est – Estonians (*n* = 47), Han – Chinese Han (*n* = 25), Man – African Mandenkalu (*n* = 23). *CGB8* has been studied only in Estonian samples (*n* = 95; Rull et al., 2008a,b).

**Fig. 3:**
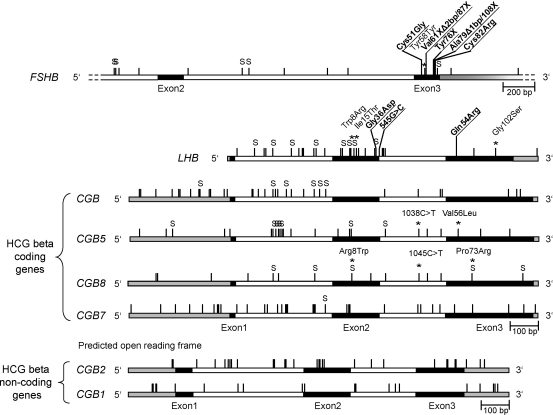
Distribution of known DNA variants across the human *FSHB* and *LHB*/*CGB* genes. The intron/exon structure of full genic regions is drawn to scale; for *FSHB* only the re-sequenced region is shown. Black, white and grey boxes represent exons, introns and untranslated regions, respectively. Vertical lines mark the positions of detected polymorphisms (Grigorova et al., 2007; Rull et al., 2008a,b; Hallast et al., 2005; Tables 2–4). Non-synonymous mutations causing clinical consequences (from Table 2) are indicated with underlined font and polymorphisms associated with a distinct phenotype (from Table 3) are marked with a star above the positions. S - singleton polymorphism detected for only one individual.

*FSHB* has been a subject to evolutionary constraints due to its unique function essential for mammalian reproduction (Wallis, 2001). The *FSHB* gene is characterized by low genetic variation and excess of polymorphisms with intermediate allele frequencies (Lamminen et al., 2005; Grigorova et al., 2007). Resequencing study in three human populations (European Estonians, Chinese Han and African Mandenkalu) identified a density of polymorphisms of 3 SNPs/1 kb (Fig. 2) (Grigorova et al., 2007). Majority of these were common polymorphisms located in non-coding regions and were shared by three human populations (Fig. 3).

In contrast to *FSHB*, the *LHB*/*CGB* genes are among the top diverse genes in the human genome (Hallast et al., 2005). The highest density of polymorphisms was detected within the genes located at the edges of the gene cluster (12.6 SNPs/kb for *LHB*, 14.6 SNPs/kb for *CGB* and 15.6 SNPs/kb for *CGB7*) (Figs. 1–3). For the central genes *CGB5* (8.1 SNPs/kb) and *CGB8* (7.5 SNPs/kb) the density of polymorphic positions was lower, but still more than twofold higher as detected for the *FSHB*. Compared to *FSHB*, the *LHB*/*CGB* genes harbour a notably higher fraction of population-specific variants. For all *LHB*/*CGB* genes the most variable region was intron 1, whereas the polymorphisms in exons have been less tolerated due to their apparent functional consequences (Fig. 3; Table 2). The variation of two HCG beta non-coding genes, *CGB2* (9.8 SNPs/kb) and *CGB1* (6.7 SNPs/kb), is not as high as reported for the rest of the gene copies. On the one hand, this could be explained by relatively shorter period for accumulation of variants due to their evolutionary younger age compared to *LHB* and *HCG beta* genes (Hallast et al., 2007). On the other hand, *CGB1* and *CGB2* are located in the central part of the *LHB*/*CGB* region, which has been shown to be less active as a gene conversion acceptor and thus mostly unaffected by its diversifying effect (see following section).

### 3.2 Role of gene conversion

**Fig. 4:**
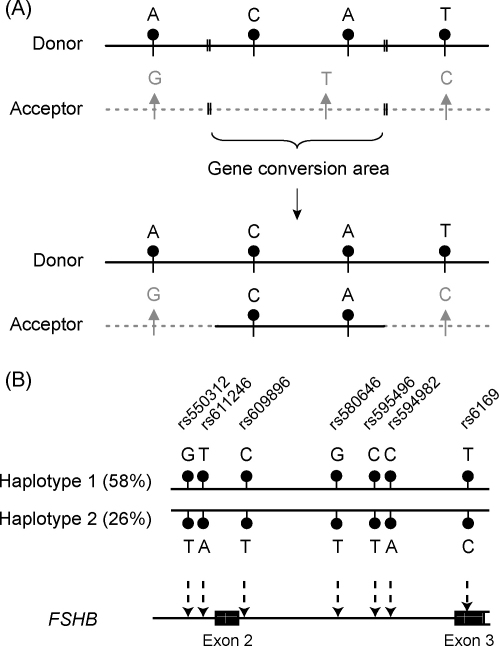
Schematic description of gene conversion and definition of haplotype variants. (A) During gene conversion the genetic material is transferred between highly identical gene copies or alleles. One locus acts as a donor of a gene conversion tract and the other as an acceptor. In case the two gene copies differ in some sequence positions within the converted tract, the variant specific to the acceptor is replaced by copying the donor-specific variant. The donor DNA sequence remains unchanged. In the figure, the donor locus is displayed as a black line with circles indicating the gene-specific positions. The acceptor locus is drawn as a grey dashed line and the gene-specific positions as triangles. (B) Definition of gene haplotypes using the two major *FSHB* gene variants as an example (Grigorova et al., 2007). Haplotypes are drawn schematically and the average frequency across European Estonians, Chinese Han and African Mandenkalu is given in brackets. The allelic composition of the two *FSHB* gene variants differs in every polymorphic position shown as black circles. The alternative alleles of the *FSHB* common polymorphisms are shown for haplotype 1 and haplotype 2. The locations of the polymorphisms within the *FSHB* gene are indicated. Rs-numbers correspond to NCBI human genome build 36 (http://www.ncbi.nlm.nih.gov/).

Gene conversion is a mechanism often found in duplicated genomic regions whereby DNA sequence information is transferred between a pair of highly identical sequences (Fig. 4A). On the one hand, it preserves the sequence similarity between duplicated gene copies of multigene families leading to concerted evolution of duplicons (Walsh, 1985; Ohta, 1991). On the other hand, it also spreads polymorphisms between homologous genes (Ohta, 1991; Huber et al., 1993; reviewed in Chen et al., 2007). Thus, when a polymorphism occurs in one gene copy, it can be transferred to another duplicated locus.

The high sequence similarity among the genes (up to 99%) as well as inter-genic regions (up to 97%) of the human *LHB*/*CGB* gene cluster provides an ideal surface for the gene conversion activity. Due to the homogenizing effect of gene conversion events, the intra-specific *LHB*/*CGB* genes have become more closely related to each other than to their counterparts in closely related species (Maston and Ruvolo, 2002; Hallast and Laan, 2009). The observed transferred sequence tracts between the *LHB*/*CGB* genes have been determined to range from a few bp to maximum 796 bp (Hallast et al., 2005). The entire gene cluster revealed the tendency of directional gene conversion: the donor loci are located in the centre and the acceptor genes at the edges of the cluster (*CGB* and *CGB7*). Consistently, the highest density of polymorphisms is mapped for the most active gene conversion acceptors, the *CGB* and *CGB7* genes (Hallast et al., 2005). However, in addition to increasing diversity of entire *LHB*/*CGB* gene cluster, gene conversion could also be the source of mutations affecting the function of the acceptor gene (reviewed in Chen et al., 2007). The process may have a functional consequence if a DNA segment is transferred from HCG beta genes to the unique *LHB* gene or from more divergent *CGB1*-like genes to the protein-coding genes in the cluster (see examples in Section 4).

### 3.3 Role of selective pressures

#### 3.3.1 *FSHB* gene

**Fig. 5:**
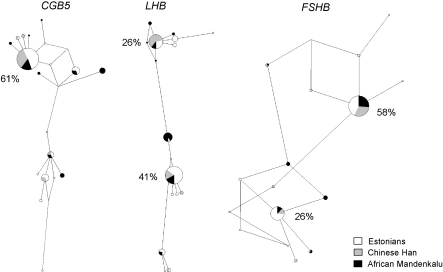
Haplotype networks for the human *CGB5*, *LHB* and *FSHB* genes. The networks were derived based on resequencing data (Hallast et al., 2005; Grigorova et al., 2007) and using the Median-Joining network algorithm implemented in NETWORK 4.201 software (http://www.fluxus-technology.com). Singleton polymorphisms were excluded from the analysis. Each node represents a single combination of SNP alleles within a gene. The size of a node is proportional to the haplotype frequency in the total dataset and the length of a line connecting two nodes correlates with the number of sequence differences between two haplotypes. The frequency of prevalent haplotypes across studied populations is given next to the major nodes.

The major factor shaping the genomic context of *FSHB* is a stringent selective pressure. No mutations, which severely affect the function of this locus, are passed through to the next generation as the carriers fail to reproduce an offspring. A closer look at the diversity patterns across the *FSHB* gene reveals a strong allelic association between all common polymorphisms that form two major gene variants called haplotypes (Fig. 4B) (Grigorova et al., 2007). A haplotype is defined as a combination of allelic variants of sequential polymorphisms, which are often inherited together. The two major *FSHB* haplotypes spread worldwide (58% and 26% of studied individuals, respectively) could be denoted as “yin-yang” gene variants since they have a contrasting composition of alleles in all common polymorphic positions (Fig. 4B). Minority of the individuals (in total 16% of subjects in three studied populations) carry rare haplotypes with distinguishing mutated positions (Fig. 5), thus the two major *FSHB* gene variants must have had a selective advantage in humans contributing to the reproductive success. Functional effects and biological consequence of the two major *FSHB* haplotypes are still to be addressed.

#### 3.3.2 *LHB*/*CGB* genes

Compared to *FSHB*, the *LHB*/*CGB* genes are evolving by a more complex scenario. The maintenance of duplicated, highly homologous *LHB* and *CGB* genes must occur through the balance between inter-locus gene conversion activity and selection targeted to the specific functions of the loci. The unique functions of LH beta-subunit are guaranteed mainly by the preservation of *LHB*-specific promoter and nucleotide positions that mediate the LH beta specificity (Henke and Gromoll, 2008). As the gene and its function are conserved in the entire vertebrate lineage, the *LHB* gene in Old world monkeys and apes must evolve under constant competition between gene conversion events and strong selective pressures (Hallast et al., 2008). In human and chimpanzee, there is evidence for Darwinian selection acting on *LHB* and the major HCG beta coding genes, *CGB5* and *CGB8* (Hallast et al., 2008). These genes provide 18–39% and 27–64% of the total HCG beta transcript pool, respectively (Bo and Boime, 1992; Miller-Lindholm et al., 1997; Rull and Laan, 2005). Still, as HCG beta coding genes are represented in the human genome by four copies, the selective pressure acting on these genes is more relaxed and facilitates accumulation of polymorphisms either by *de novo* substitutions or gene conversion events. In three populations representing Africa, Asia and Europe, the *CGB5* and *LHB* genes comprised of one (61% of individuals) or two (67% of individuals) major haplotypes, respectively (Fig. 5). The two major haplotypes in *LHB* differ from each other in seven out of 16 identified polymorphic positions. In contrast, *FSHB* has two completely different major haplotypes with no shared alleles. For all *HCG beta* genes 30–61% of studied individuals were carrying various minor gene variants.

## 4 Genetic variation of human gonadotropin beta genes

A few mutations that affect hormonal function and metabolism have been characterised in the single-copy *LHB* and *FSHB* genes. As an analysis of the multi-copy *CGB* genes is technically challenging and the phenotypic effect of mutations is probably weaker due to redundancy, the mutational spectrum of these genes is not as extensively studied. In addition to mutations, a list of polymorphisms has been found to be associated with specific phenotypes.

### 4.1 Gonadotropin beta gene mutations with known functional effects

In total five mutations in *FSHB* have been reported in three male and six female patients, all having a severely impaired sexual development and infertility (Table 2; Fig. 3; Figure S1). Three mutations, Val61Δ2 bp/87X, Tyr76X and Ala79Δ1 bp/108X in exon 3 lead to a premature stop codon and truncation of the FSH beta protein (Matthews et al., 1993; Layman et al., 1997; Phillip et al., 1998; Kottler et al., 2010). Two other identified mutations in exon 3, Cys51Gly and Cys82Arg, alter a cystein knot structure of the FSH beta peptide. The cystein knot is crucial for hormone dimerization and bioactivity (Layman et al., 1997; Lindstedt et al., 1998).

Three mutations have been described in the *LHB* gene: Gly36Asp, Gln54Arg and a substitution G to C at position 545 (from transcription start) in intron 2 (Table 2; Fig. 3) (Weiss et al., 1992; Valdes-Socin et al., 2004; Lofrano-Porto et al., 2007). The latter mutation disrupts splicing of intron 2 and results in an insertion of 236 bp into *LHB* mRNA sequence and a frameshift in exon 3 (Lofrano-Porto et al., 2007). As LH is not necessary for sexual differentiation before birth, all patients had a normal phenotype at birth, including descended testes in males (Hughes, 2001). Clinical signs caused by the lack of bioactive LH appear after pubertal age: delayed puberty and arrested spermatogenesis in males, secondary amenorrhoa and infertility in a female patient (Table 2).

### 4.2 Polymorphisms associated with phenotypic traits

#### 4.2.1 *FSHB* gene

Among the three common synonymous changes described in the coding region of *FSHB*, the 2623T>C (Tyr58Tyr) in exon 3 has been demonstrated to be more frequent among obese patients with polycystic ovary syndrome (PCOS) than in healthy females (Table 3; Fig. 3; Figure S1) (Tong et al., 2000).

A substitution G>T located in *FSHB* promoter region at position −211 from mRNA transcription start site has originally been described in a male patient with azoospermia and isolated FSH deficiency (Mantovani et al., 2003). This SNP is located within a progesterone response element (PRE) conserved among numerous placental mammals and capable of enhancing the gene transcription up to 9-fold (Webster et al., 1995). A cohort of young European men (*n* = 554) showed an association between the minor allele T carrier status and reduced serum FSH level (Grigorova et al., 2008). Compared to the wild-type homozygotes (GG), the heterozygotes (GT) and the homozygotes (TT) for the minor allele had on average 15.7% and 40% lower levels of FSH in their bloodstream, respectively. The association with lower male serum FSH level was further confirmed in a cohort of men diagnosed with infertility (*n* = 1029) (Grigorova et al., 2010). Moreover, the minor allele of this polymorphism was shown to be overrepresented among infertility patients. *FSHB* −211G/T is the first described polymorphism shown to significantly affect the male serum FSH levels.

#### 4.2.2 *LHB* gene

The most studied variation in *LHB* gene is a combination of two amino acid changes that are always found together as a haplotype on one chromosome: Trp8Arg/Ile15Thr (Table 3; Fig. 3; Figure S1) (Furui et al., 1994; Okuda et al., 1994). Trp8Arg substitution is mainly responsible for an altered immunoreactivity of the hormone and Ile15Thr substitution introduces an extra glycosylation site into the altered LH beta peptide. The carrier frequency of the variant allele (V-LH beta) differs widely between ethnic groups (0–50%) (Nilsson et al., 1997; Lamminen and Huhtaniemi, 2001). Compared to normal LH, the hormone formed by the V-LH beta possesses an increased *in vitro* biopotency and altered half-life in circulation, although the published data on the length of the half-life is contradictory (Manna et al., 2002; Wide et al., 2010). There are numerous published studies addressing the carrier status of the V-LH beta variant in relation to various clinical conditions (Table 3). The variant LH form has been suggested to suppress gonadal function and to be involved in the development of subfertility (Furui et al., 1994; Haavisto et al., 1995; Lamminen and Huhtaniemi, 2001). The V-LH beta variant could have originated by an ancient gene conversion event where one of the *CGB* genes has acted as a donor and *LHB* as an acceptor (see Section 3.2; Hallast et al., 2005). The concerted substitutions (443T>C and 465T>C) in V-LH exon 2 leading to Trp8Arg/Ile15Thr changes correspond to the nucleotide sequence in the respective positions of all six *CGB* genes.

Additionally, Gly102Ser substitution in exon 3 has been found to be associated with reproductive disorders in some populations (Ramanujam et al., 2000).

#### 4.2.3 *CGB* genes

A resequencing study of the *CGB5* and *CGB8* genes detected three rare non-synonymous substitutions (Val56Leu in *CGB5* and Arg8Trp, Pro73Arg in *CGB8*) only among recurrent miscarriage patients as possible variants increasing the risk of recurrent pregnancy loss (Table 3; Fig. 3; Figure S1) (Rull et al., 2008b). Similarly, a rare T>A mutation in *CGB8* located adjacent to an initiator element for HCG beta transcription (4 bp before transcription start site) was identified as a potential risk variant in developing a recurrent miscarriage (Rull et al., 2008b). A significant protective effect toward recurrent miscarriage was associated with two SNPs located at identical positions in intron 2 in both, *CGB5* (1038C>T) and *CGB8* (1045C>T), and with four *CGB5* promoter variants (−155G>C/−147G>del/−144T>C/−142T>A) (Table 3) (Rull et al., 2008b). These variants reduced the risk of recurrent miscarriages up to 1.8-fold. The haplotype-forming alternative *CGB5* promoter variant may have arisen by the transfer of a gene conversion tract derived from the *CGB8* promoter sequence as a donor.

### 4.3 Non-synonymous amino acid substitutions with unknown phenotypic consequence

Several additional variants have been reported in the human *FSHB*, *LHB* and *HCG beta* coding genes that cause a non-synonymous substitution, but the data about the phenotypic consequences in the study participants is unavailable (Table 4; Figure S1) (Jiang et al., 2002; Hallast et al., 2005). The Ala-3Thr change in the signal peptide of LH beta has been shown to cause different *in vitro* signal transduction properties compared to a wild-type preprotein presumably due to altered conformation of secreted LH (Jiang et al., 2002). The alternative signal transduction pathway apparently prevents desensitisation of LH/HCG receptor and therefore supports the continuous function of *corpus luteum* during gestation (Cameo et al., 2004). The polymorphism leading to Val79Met substitution in the exon 3 of *CGB5* was shown in *in vitro* experiments to code for the altered protein unable to fold correctly and assemble with alpha-subunit (Miller-Lindholm et al., 1999). Individuals carrying this polymorphism could possibly have a subtle deficiency of bioactive HCG but its association with an impaired reproductive outcome has not been established.

## 5 Comparative expressional profile of human gonadotropin beta genes

Pituitary FSH and LH are produced in a pulsatile manner with differential frequencies and amplitude regulated by gonadotropin releasing hormone (GnRH) (reviewed in Marshall et al., 1993; Miller et al., 2002). On the other hand, *CGB* genes are expressed and HCG produced continuously in placenta over two alternative signal transduction pathways in order to avoid receptor desensitisation (Cameo et al., 2004). Rate-limiting for the respective hormone production is the transcription of *FSHB*, *LHB* and *HCG beta* coding genes. Thus, the genetic and genomic context of the *FSHB*, *LHB* and *HCG beta* genes affects the profile of the hormone production.

For the *FSHB* gene at least four alternative transcripts have been described that encode an identical unmodified protein. Different transcripts arise due to combinations of alternate splicing of the first non-coding exon and at least two potential polyadenylation signals (Jameson et al., 1988). In case of *LHB* and *HCG beta* coding genes, no splice variants have been identified. Although alternative transcripts have been reported for the *HCG beta* non-coding genes *CGB1* and *CGB2*, their functional effect is yet to be determined (Bo and Boime, 1992; Berger et al., 1994; Rull and Laan, 2005).

### 5.1 Profile of FSHB and LHB gene expression

**Fig. 6:**
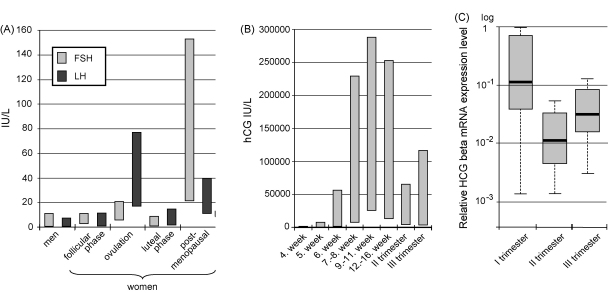
Normal ranges of (A) serum FSH and LH concentrations in men and women; (B) serum HCG dynamics during pregnancy and (C) levels of *HCG beta* mRNA transcripts in placental tissue. The graphs (A) and (B) are drawn based on reference values of United Laboratory of University of Tartu Clinics, Estonia. The graph **C** was drawn based on the data from Rull et al. (2008a,b). The boxes represent the 25th and 75th percentile and median is denoted as the line that bisects the boxes.

Pituitary LH and FSH have been preserved during evolution as functionally critical hormones that guarantee successful reproduction and survival of a species. Thus, large-scale fluctuations in the transcriptional activity of the *FSHB* and the *LHB* genes might be fatal for the reproductive success. An optimal concentration of *LHB* and *FSHB* transcripts and the coded protein is kept strictly in narrow ranges in men as well as in women during follicular and luteal phases, but undergoes a rise during ovulation and post-menopausal period (Fig. 6A) (Owen, 1975; Sherman and Korenman, 1975; Zumoff et al., 1982; Baird, 1983). Overexpression of *FSHB* could cause polycystic ovary syndrome as in female FSH overexpressing mice, whereas severe reduction in *FSHB* transcription and FSH production could cause infertility in females or poor sperm quality in males (Kumar et al., 1997, 1999; Plant and Marshall, 2001). Large fluctuations in *LHB* expression could lead to hypogonadism, infertility and several other endocrine disorders in both sexes as can be seen in patients with various mutations in the *LHB* gene (Tables 2 and 3) (reviewed in Huhtaniemi and Themmen, 2005).

Serum FSH levels depend on the selective regulation of *FSHB* mRNA transcription by the action of pituitary ja gonadal factors such as activin and inhibin, while the expression of glycoprotein alpha-subunit (*CGA*) mRNA remains unaffected (Carroll et al., 1989; Farnworth, 1995; Lin and Ge, 2009). The critical role of the *FSHB* transcription in determining FSH levels was also confirmed by mapping of a polymorphism in a regulatory region −211 bp upstream of *FSHB*. The alternative allele of this polymorphism reduces the *FSHB* transcription by 46–58% and leads to lower FSH production in men (Table 3) (Hoogendoorn et al., 2003; Grigorova et al., 2008, 2010). In case of *LHB*, several variants in the promoter region of V-LH beta have been identified, which increase expressional activity and alter the response to hormonal stimulation (Jiang et al., 1999).

### 5.2 Profile of HCG beta gene expression

In contrast to pituitary gonadotropins, placental HCG beta mRNA is expressed by four duplicate genes and larger inter-individual differences in the total amount of produced mRNA and in hormone levels are tolerated (Miller-Lindholm et al., 1997; Rull and Laan, 2005; Rull et al., 2008a). The highest variability in hormone levels is seen during the I trimester when HCG concentration doubles every 48–72 h and causes up to ∼11-fold inter-individual differences (Fig. 6B) (Hay, 1988). Large fluctuations in gene expression probably reflect high genetic variation in the *HCG beta* genes (see Section 3.1). Transcriptional activity of the *HCG beta* genes changes through the course of a pregnancy and in correlation with the dynamics of the HCG hormone (Fig. 6B and C).

Although wide ranges in HCG beta transcriptional activity are tolerated during the pregnancy, biparental expression of *CGB* genes is required in order to provide a necessary amount of HCG beta-subunits and avoid complications in early pregnancy (Uusküla et al., unpublished data). Too low mRNA expression could lead to miscarriage and excessively high amount of HCG beta transcripts could be a marker for ectopic or molar pregnancy (Rull and Laan, 2005; Rull et al., 2008a).

### 5.3 Tissues of minor expression of gonadotropin beta genes

In addition to major sites of expression – pituitary and placenta – gonadotropin hormone production in minor concentrations has been reported in other tissues. An ectopic production of pituitary gonadotropins LH and FSH in normal non-malignant tissues is limited; minimal amount of *LHB* transcripts have been detected in testis and placenta (Berger et al., 1994; Giovangrandi et al., 2001). Both hormones have been detected in tumours that arise from gonadotroph cells of the pituitary gland. Gonadotroph adenomas account for approximately 40% of all clinically recognized macroadenomas and approximately 80% of surgically excised clinically non-functioning adenomas (Valimaki et al., 1999). In these adenomas the glycoprotein hormone beta-subunits may be detected by molecular, immunological or immunohistochemical staining. The clinical manifestation is delayed due to inefficient secretion of the hormone and its subunits (Jaffe, 2006; Valimaki et al., 1999).

*HCG beta* genes have been reported to be expressed in minimal amount in normal non-trophoblastic tissues, mostly in testis, prostate, thymus, skeletal muscle and pituitary gland (Bellet et al., 1997; Reimer et al., 2000). The role of HCG in normal tissues may be related to autoregulatory mechanisms, beta-subunit of the hormone has been shown to exert growth-promoting activity (Butler and Iles, 2004). HCG probably acts in an auto-/paracrine way, a transcrine pathway has been suggested for the testis, prostate and uterus (Berger et al., 2007).

Several non-trophoblastic tumors, like bladder, renal, prostate, lung, gastrointestinal, neuroendocrine, breast and gynecological cancers, have been shown to produce HCG beta-subunit and to a lesser extent intact HCG (Stenman et al., 2004). The role of HCG in the carcinogenesis could be associated with enhancement of invasion, angiogenesis, inhibition of apoptosis and escape from immune surveillance (Butler and Iles, 2004; Herr et al., 2007; Reisinger et al., 2007). The same biological means are used by trophoblasts to ensure successful implantation and placentation. At the genomic level, activation of transcription of *CGB*, *CGB5* and *CGB8* genes has been associated with malignant transformation of non-trophoblastic cells (breast, bladder, prostate, thyroid, testis and renal cancer) (Bellet et al., 1997; Giovangrandi et al., 2001; Hotakainen et al., 2007).

## 6 Conclusive remarks

The gonadotropin beta-subunit genes are functionally related and have a common evolutionary ancestry. Nevertheless, the single-copy *FSHB* and the multi-copy *LHB*/*CGB* genes exhibit locus-specific differences in their genomic context and its evolution, genetic variation and expressional profiles. The *FSHB* gene is conserved in vertebrates, maps to a gene-poor region and is evolving under strong selection due to its conserved function. Any non-synonymous change has an immediate phenotypic consequence impairing the reproductive potential of the carrier. In contrast, the primate-specific *LHB*/*CGB* gene cluster is located in a dynamic gene-rich region and its evolution is driven by the constant interplay between gene conversion and selective forces. Gene conversion is also the major force, which has raised the *LHB*/*CGB* genes among the most highly polymorphic genes in the human genome. Transcription of the pituitary *FSHB* and *LHB* genes is rate-limiting in the hormone production and kept in a narrow expressional window. In contrast, placental *HCG beta* mRNA is expressed by four duplicate genes that exhibit high genetic variation. Thus, large inter-individual and inter-genic fluctuations are tolerated regarding the total amount of produced mRNA and hormone levels.

## Figures and Tables

**Table 1:** Characteristic features of gonadotropin specific beta-subunits.

	FSH	LH	HCG
Protein			
Mature β-subunit protein	111 amino acids	121 amino acids	145 amino acid
Time of production	Postnatal	Postnatal	Prenatal
Production pattern	Pulsatile	Pulsatile	Continuous
Biologic half-life	3–4 h	20–30 min	∼24 h
Main function	Stimulates and regulates spermatogenesis in men and follicular maturation in women	Stimulates steroidogenesis in testicular Leydig cells in men and promotes ovulation in ovarian luteal cells in women	Promotes implantation and placentation, and production of progesterone during pregnancy, stimulates sexual differentiation of the male fetus
Receptor	FSHR	LH/CGR	LH/CGR

Gene			
Specific β-subunit coding gene	*FSHB*	*LHB*	*CGB*, *CGB5*, *CGB8*, *CGB7*
Chromosomal localisation	11p13	19q13.32	19q13.32
Gene length	4262 bp	1111 bp	1467 bp
Major site of expression	Anterior lobe of pituitary gland	Anterior lobe of pituitary gland	Syncytiotrophoblastic cells in placenta
Alternative splice forms	Yes	No	No

**Table 2:** Mutations in the human gonadotropin beta-subunit genes with a clinical phenotype.

Nucleotide substitution^a^	Amino acid change^b^	Male phenotype	Female phenotype	Bioactivity/effect at protein level	Reference
*FSHB* mutations					
2600T>G exon 3	Cys51Gly		Primary amenorrhea, infertility, retarded sexual development (one case)	Aberrant tertiary structure of the protein, loss of bioactivity	Layman et al. (1997)
2631TG>del exon 3	Val61Δ2bp/87X	Azoospermia, hypogonadism, very low FSH (one case)	Primary amenorrhea, infertility, retarded sexual development (two cases)	Loss of bioactivity, truncated protein	Matthews et al. (1993), Layman et al. (1997) and Phillip et al. (1998)
2677C>A exon 3	Tyr76X	Azoospermia, hypogonadism, mild gynecomastia (one case)	Primary amenorrhea, partial breast development (two cases)	Loss of bioactivity, truncated protein	Layman et al. (2002), Lofrano-Porto et al. (2008) and Berger et al. (2005)
2684G>del exon 3	Ala79Δ1bp/108X		Primary amenorrhea, impaired pubertal development (one case)	Loss of bioactivity, truncated protein	Kottler et al. (2010)
2693T>C exon 3	Cys82Arg	Azoospermia, hypogonadism, very low FSH (one case)		Aberrant tertiary structure of the protein, loss of bioactivity	Lindstedt et al. (1998)

*LHB* mutations					
528G>A exon 2	Gly36Asp	Impaired spermatogenesis, hypoplastic Leydig cells (one case)		Absent	Valdes-Socin et al. (2004)
545G>C intron 2		Azoospermia, hypogonadism (two males^c^)	Secondary amenorrhea, infertility (one female^c^)	Absent, abnormal splicing of mRNA	Lofrano-Porto et al. (2007)
818A>G exon 3	Gln54Arg	Absence of spontaneous puberty, low testosterone level (one case)		Absent, no binding to receptor	Weiss et al. (1992)

a Nucleotide positions are defined relative to the transcription start site on the genomic DNA sequence; GenBank references: NC_000011.8 for *FSHB*, NC_000019.8 for *LHB*/*CGB*/*CGB5*/*CGB8*/*CGB7* (Supplementary Figure S1).

b Amino acid positions are defined based on the sequence of a mature protein.

c Consanguineous siblings.

**Table 3:** Variants in the human gonadotropin beta-subunit genes associated with phenotypic traits.

Nucleotide substitution^a^	Amino acid change^b^	Assessed phenotype	Number of carriers/total number of studied individuals	Association	Reference
Variants in *FSHB*					
−211G>T 5′upstream		Azoospermia, FSH deficiency	One male patient with normal virilization	na	Mantovani et al. (2003)
		Serum FSH concentration	131/554 cohort of young men	Yes – minor allele carriers had lower FSH level	Grigorova et al. (2008)
			283/1029 male partners of infertile couples		Grigorova et al. (2010)
2623T>C exon 3	Tyr58Tyr	Polycystic ovary syndrome (PCOS)	89/135 female patients	Yes	Tong et al. (2000)
			54/105 normal control females		

Variants in *LHB*					
443T>C/465T>C exon 2	Trp8Arg/Ile15Thr (v-LH)	PCOS	32/153 female patients	No	Rajkhowa et al. (1995)
			31/212 healthy females		
		PCOS	1/30 female patients	No	Elter et al. (1999)
			5/30 healthy females		
		PCOS	46/328 female patients	No	Tapanainen et al. (1999)
			169/881 control females		
		Subfertility/infertility	3/3 female patients	na	Furui et al. (1994)
			0/2 fertile females		
		Infertility	16/97 female patients	Yes	Takahashi et al. (1998)
			14/169 fertile females		
		Infertility	12/145 male patients	No	Ramanujam et al. (2000)
			15/200 fertile males		
		Delayed tempo of pubertal progression	13/49 healthy boys	Yes - v-LH carriers had smaller testicular volume, slower growth rate	Raivio et al. (1996)
		Endocrine disorder and/or gynecologic disease	45/245 female patients	Yes	Takahashi et al. (1999)
			13/153 fertile females		
		Menstrual disorders	21/176 female patients	No	Ramanujam et al. (1999)
			20/200 normally ovulating females		
		Endometriosis	16/85 female patients	No	Gazvani et al. (2002)
			29/145 females with unknown fertility		
		Male eunucoid syndrome	One fertile male patient	na	Shiraishi and Naito (2003)
		Non-obstructive male infertility	12/95 male patients	No	Lee et al. (2003)
			29/200 fertile males		
961G>A exon 3^c^	Gly102Ser	Infertility, endometriosis, PCOS	2/52 female patients	No	Liao et al. (1998)
			0/212 healthy females		
		Menstrual disorders	7/176 female patients	Yes	Ramanujam et al. (1999)
			0/200 normally ovulating females		
		Infertility	5/145 male patients	Yes	Ramanujam et al. (2000)
			0/200 fertile males		

Variants in *CGB5*					
−155G>C/−147G>del/		Recurrent miscarriage (RM)	26–27/184 RM couples	Yes	Rull et al. (2008b)
−144T>C/−142T>A 5′upstream			45–48/195 fertile females		
1038C>T intron 2		RM	30/184 RM couples	Yes	Rull et al. (2008b)
			51/195 fertile females		
1178G>C exon 3	Val56Leu	RM	1/184 RM couples	na	Rull et al. (2008b)
			0/195 fertile females		

Variants in *CGB8*					
806C>T exon 2	Arg8Trp	RM	1/184 RM couples	na	Rull et al. (2008b)
			0/195 fertile females		
1045C>T intron 2		RM	1/184 RM couples	Yes	Rull et al. (2008b)
			7/195 fertile females		
1237C>G exon 3	Pro73Arg	RM	1/184 RM couples	na	Rull et al. (2008b)
			0/195 fertile females		

a Nucleotide positions are defined according to the location of the transcription start site in the genomic DNA sequence; GenBank references: NC_000011.8 for *FSHB*, NC_000019.8 for *LHB*/*CGB*/*CGB5*/*CGB8*/*CGB7* (Supplementary material, Figure S1).

b Amino acid positions are defined based on the sequence of a mature protein.

c Originally described as 1502G>A; na – statistical tests were not applicable.

**Table 4:** Non-synonymous amino acid substitutions with unknown phenotypic consequence.

Gene	Position relative to mRNA^a^	Position relative to ATG^b^	Amino acid change^c^	Reference
*FSHB*	946G>T		Ser2Ile	Cargill et al. (1999)

*LHB*	406G>A	397	Met-6Ile	Hallast et al. (2005)
	413G>A	404	Ala–3Thr	Jiang et al. (2002)
				Hallast et al. (2005)
	450A>G	441	His10Arg	Hallast et al. (2005)

*CGB*	1363A>C	998	Asp117Ala	Hallast et al. (2005)

*CGB5*	794G>A	429	Arg6Gln	Hallast et al. (2005)
	1247G>A		Val79Met	Miller-Lindholm et al. (1997)
	1362A>C	997	Asp117Ala	Hallast et al. (2005)
*CGB8*	869G>A		Val29Ile	Rull et al. (2008b)

*CGB7*	782G>A	417	Arg2Lys	Hallast et al. (2005)
	787/788AT>CC	422/423	Met4Pro	
	1162G>A	797	Ala51Thr	
	1232C>T	867	Arg74Cys	
	1363C>A	997	Ala117Asp	

a Nucleotide positions are defined according to the location of the transcription start site in the genomic DNA sequence; GenBank references: NC_000011.8 for *FSHB*, NC_000019.8 for *LHB*/*CGB*/*CGB5*/*CGB8*/*CGB7* (Supplementary material, Figure S1).

b Translation start site as in original reports; GenBank references: NC_000011.8 for *FSHB*, NC_000019.8 for *LHB*/*CGB*/*CGB5*/*CGB8*/*CGB7* (Supplementary material, Figure S1).

c Amino acid positions are defined based on the sequence of a mature protein.
