# Supplementary material for: Genomics and genetics of gonadotropin beta-subunit genes: Unique FSHB and duplicated LHB/CGB loci
Source: Mol Cell Endocrinol. 2010 Nov 25;329(1-2):4–16. doi: 10.1016/j.mce.2010.04.024 (PMC2954307; doi:10.1016/j.mce.2010.04.024)
Supplement: Supplementary file 1 [file mmc1.doc]

**Genomics and genetics of gonadotropin beta subunit genes: unique *FSHB* and duplicated *LHB/CGB* loci**

**Authors:**

Liina Nagirnaja, Kristiina Rull, Liis Uusküla, Pille Hallast, Marina Grigorova and Maris Laan

## Supplementary material

**Figure S1.** Reference sequences for *FSHB*, *LHB*, *CGB*, *CGB5*, *CGB7* and *CGB8* genes with mutations and polymorphisms from Tables 2-4. Genbank reference sequences are given in the orientation of transcription and from transcription start site (NCBI human genome build 36.3; http://www.ncbi.nlm.nih.gov/). Translations start site (ATG), polymorphic positions and amino acid changes are highlighted based on Table 2, Table3 and Table 4.

**Figure S2.** Aminoacid sequence alignment for the products of hCG beta-subunit coding genes (*CGB, CGB5, CGB7, CGB8*), *LHB*, and *CGB1* and *CGB2*. The comparison is based on the 132 aminoacid sequence predicted for *CGB1* and *CGB2* (Bo and Boime 1992; Dirnhofer et al. 1996). However, this transcript may not be the most abundant at mRNA level (Rull and Laan 2005).

**Figure S1.** Genbank reference sequences for *FSHB*, *LHB*, *CGB*, *CGB5*, *CGB7* and *CGB8* genes with mutations and polymorphisms from Tables 2-4.

## Homo sapiens follicle stimulating hormone, beta polypeptide (*FSHB*), 11p13

Genbank Reference sequence NC_000011.8; GI:51511727. Chromosome region from 30209139 to 30213400 base; 4262bp

ACAGCTCTTGCCAGGCAAGGCAGCCGACCACAGGTGAGTCTTGGCATCTACCGTTTTCAAGTGGTGACAGCTACTTTTGAAATTACAGATTTGTCAGGACATGGAGGACAAAACTAGAGCTTCTCACTACTGTTGTGTAGGAAATTTATGCTTGTCAACCTGGCTTGTAAAATATGGTTAATATAACGTAATCACTGTTAGCAAGTAACTGACTTTATAGACCAATATGCCTCTCTTCTGAAATGGTCTTATTTTAAACAAATGTGAGCAAAAGAAAATATTTATGAGATTCTAAAAATGAAGACATAATTTTGTAGTATAGAATTTTCTTGGCCAGGAATGGTGGCTCATGCTTGTAATCCCAGCACTTTGGGAGGCCAAGGTCAGAGGATTGCTTGAGCCTGGAAGGTTGAAGATGCAGTGATTCATGATTATACCACTGCACTCCAGCCTGGGCAACAGAGCAAGACCCTGTCTCAAGAAAAGAAAAGAATTTTATTTTTCTTTTCAGACAAAAATAGACTTTAAAATAATAATGGAAGAACAAATATGATGATCACAATTATCAGAGTAATTACTTTATGACAGTCAGCAATAAGATTCTAATCTTTAAATATTCCTCTGCTTAAATCATTATATTGGAGTTTTGATCTATAATATATTCCCACCCTGACCCAAAAATTGAAGAAGGACAAGGAAAAATGTTGTTCCAAGAAACAAAGATGTAAGTAAAAAGGCATAAGGAAGGAAAAAAAACTTTTGAAGCAAAATGTGATTGAGGAGGATGAGCAGACCAATTATTTTTGGTTTGGTCAGCTTACATAATGATTATCGTTCTTTGGTTTCTCAGTTTCTAGTGGGCTTCATTGTTTGCTTCCCAGACCAGGATGAAGACACTCCAGTTTTTCTT

###### Ser2Ile

**946G>T**

CCTTTTCTGTTGCTGGAAAGCAATCTGCTGCAATAGCTGTGAGCTGACCAACATCACCATTGCAATAGAGAAAGAAGAATGTCGTTTCTGCATAAGCATCAACACCACTTGGTGTGCTGGCTACTGCTACACCAGGGTAGGTACCATGTTTTGCTGGAAGCAAGGGTGTTGAAGGTCTGTATTAGGCCGGTTTCATTAGTTTCTACTTTATCAATATTTTATGTATTCTAAGTAACAGCCATGAGTCCTTTAGCCAAGACTGTCTGTGTTGTGATTGGGGTTAATGACCACGATATCACTTAGATGTTTGGGCTTGGATTTGATTTGGGTAAATTTAGGAAAGCCTCAGATTTAATCTGATCAATTTGGTACTAGTCCAACTTTGCATCTACAGGGAAAAAGTATTTCTATGTTACGTTTTTACACATAGAGAGATAAACATGGAAACATACATATATTTAATCATAAAGGACCTATAATATTCTCATAAAGGCAATTTCTTTAACTGACACTACATCTTTGACACAAAATCACACCAAAATATGTCTCCAAGTCACATAAAAACATAGACAGCCACTTAAAAAAATTGTCTTCCTGGCCCTACTAAATACAAATGCCAAAAAACAGCCTGAGAACACAATCAATTCTTGCAGACTGTTAGAACAAAAATGAATCAGCAAACCCACTCCCTTCGTTATAGCATTGAGAAAACCAAGACATAGAGGCATCAGTTGCTAGTCTGTGTTTGCAGTTTCCTTGCATTAATACAAGTAGAGAAATAGTTTCCATGGTGCTTTCTTTTTTCTCTGCAGCACCCCTAATTATCTATGCAGAATTTCATTCTATAAACTAAAATTGAAAATGGCAACTTTTTAAATGAACGATACTTTATTTGACGGTAAATGAGTTTGATCAAACTCCATTTATTACACAATTTATTGCACCTTCTTGGGATATACATTTGGTAGGATGATATTAAAATAAACAGAAGCCCCAATTTCTCTACGCAGTATAAATAATTTTTCCACTGGAAAGTGCTACTACAAATAATTTCTACCTGGATTAAAAATTCTTATATGCAAACTGCATATCCTTTGAAACTAGGAACCCTGCAAAGTATACAGCTTTCAAGGGAGAAAAATGTCCACAAGGAGTTGGAATATTTAAAATCTTATGTTAGCCTTAGCAAACATGTTAACTTAAGCATTAAAATTTAAAATTATATATTTTTGACCTTTTATAAATACTCAGGGCAGTGTATTTTAAAATATTTTTTCTGAGACATTGGATATCTTTGTTTATGGTTTGTTATTAATACAGCTTTCAATTAAATATGAAAAGTCAACTTAAAATCCTGTCATGTTTTTCATCATTTTTCTATGCTAAAATTCAAAGTTCCTTTATATTTTGAAAAATAGTTAATATTTTGATATAGCCATAGGAAGTAAGAAAAGAAATTACTTGTATTTTCTGGAAGATTTCAAGAACAATTTAGAAATGTAAATAGCATATAGGTCATTTATGAGGTCATGTTTTAATGGGTAAATGTTAGAGCAAGCAGTATTCAATTTCTGTCTCATTTTGACTAAGCTAAATAGGAACTTCCACAATACCATAACCTAACTCTCTTCTTAAACTCCTCAGGATCTGGTGTATAAGGACCCAGCCAGGCCCAAAA

**Cys51Gly Tyr58Tyr Val61Δ2bp/87X**

**2600T>G 2623T>C 2631TG>del**

TCCAGAAAACATGTACCTTCAAGGAACTGGTATACGAAACAGTGAGAGTGCCCGGCTGTGCTCACCATGCAGATT

**Tyr76X Ala79Δ1bp/108X Cys82Arg**

**2677C>A 2684G>del 2693T>C**

CCTTGTATACATACCCAGTGGCCACCCAGTGTCACTGTGGCAAGTGTGACAGCGACAGCACTGATTGTACTGTGCGAGGCCTGGGGCCCAGCTACTGCTCCTTTGGTGAAATGAAAGAATAAAGATCAGTGGACATTTCAGGCCACATACCCTTGTCCTGAAGGACCAAGATATTCAAAAAGTCTGTGTGTGTGCAATGTGCCCAGGGGACAAACCACTGGATCAGGGGATTCAGACTCTACTGATCCCTGGTCTACTGGCAGAGGGAACTCTGGGAATTGAGAGTGCTGGGGGCCAGGACTCCATCATGATTCAGCTCTATATTCCTAGGTCTGATTTCATAAGGTTTATTCAGTCTTAACTCACAGACTTGTGCCTGGTTTCTTCTTTAAAAATCTTAGAAATCTTCTCAGGCAATGCCTCTCTCTTAGGGGGAAACATAAGCCTAGAAGGAGGAAGCAGTAATGGGAGTGAGTGAAAGAACTAACTGCAGCAGTCTTCTGGTAGACTCTTGGGCCCTCTAGAGCAAGGTCAGCATCTTCAGCATTGTAGCGTCAATGCCTAGCACTCTGCCTGGAACTTAGAAACACAACAATGACTTCTTTAGATCAGAAAGGTCAAGGGTAGAAAATACTGGAAGACGATGTTTGAGGTAAGCTGATGAGGCTGCCCGCAGCCACACCAGTCCCATGAAAGTTAGTGGCATCAGTTCCACCTCGCCTTTTCTCCAGCACATGGAGTATTGAGACATGATGTATCTTTCTGAATTGTTTGGTACAGATGGGGAGTAACAGAGCTCAAGATTTCCAAGCTATTACTACCAAGCCTGTTAGTTAAGGGCAAAGGCAAGAAATTGTAATTTGGGGCTGTGGAAATTAGCCTGCCTCTATTCATTACTTAAACAAATTGATCACATGCTACTAGGCTCCTGCAAAACTCCTTTTTGAGATAAAGGGAAAAAACCAAACTATCTCACCCTACCCTCCCTAGGATCCACTTCTTTGGAATGACAAAGGATTTGAAAGTAGGTTTGAAAGCAGTTTCAGCAATTTAATAAATATAATTAATTTGTCTACAAATATATTTGTATAAATAAATAGCTCCTTTAGAAAGAATTAGCCATGGGGGACGAGGGGAAACTGCTGTTTTCTAGGATCCTGTCTACATCAATCTTCTATTTTATCCATCCATGTTCTCCCAAATCTGTGCTTTCTTTCAACAGGTTATATATTAAAACTATTTCATGAGTTGATTTCTTTTAAACGTGTTAACTGTCTTAGTTATGCACTCAGTTTCACACTCATATTGTTTAACTAATTTATTTAAATCTTATTTTTTTAATAAAGATGCTAGCCACCAGAGTCACAGGCTTGGATTGTTTTATGTACAAACAGATGACTTAGATATTCTGTATTTTATAATATTAGTGGAATGAAATCTTAAAATATAATTCCCAGTGTTTCTATAAATATTACCTTTCCTTATCTTTGGAGATATTAAAAATAATTTTGTTGGATTTCTGAAGTGTTTTGTCACTTAAATTTCCTGTCATTTTTTGAAGACATTTTCTGATGTAATTTGGGAGAAAAAAAGCATAGA

## Homo sapiens luteinizing hormone, beta polypeptide (*LHB*), 19q13.32

GenBank Reference sequence NC_000019.8; GI:42406306. Chromosome region from 54211049 to 54212159 base; 1111bp

####

GCACCAAGGATGGAGATGCTCCAGGTAAGACTACAGGGCCCCTGGGCACCTTCCACCTCCTTCCAGGCCATCACTGGCATGAGAAGGGGCAGACCCGTGTGAGCTGTGGAAGGAGGCCTCTTTCTGGAGGGGCATGACCCCCAGTAAGCTTCAGGTGGGGCAGTTCCTGAGGGTGGGGATCTGAAATGTTGGGGCATCTCAGGTCCTCTGGGCTGTGGGGTGGGCTCTGAAAGGCAGGTGTCCGGGTGGTGGGTCCTGAATAGGAGATGCCGGGAAGGGTCTCTGGGTCTTTGTGGGTGGTGTACCACGCGGGATGGGAAGGCCAGGACTCGGGGCTGCGGTCTCAGACCTGGGTGAAGCAGTGTCCTTGTCCCA

**Met-6Ile Ala–3Thr Trp8Arg** **His10Arg**

**406G>A 413G>A 443T>C 450A>G**

GGGGCTGCTGCTGTTGCTGCTGCTGAGCATGGGCGGGGCATGGGCATCCAGGGAGCCGCTTCGGCCATGGTGCCA

**Ile15Thr**

**465T>C**

CCCCATCAATGCCATCCTGGCTGTCGAGAAGGAGGGCTGCCCAGTGTGCATCACCGTCAACACCACCATCTGTGCC

**Gly36Asp**

**528G>A 545G>C**

GGCTACTGCCCCACCATGGTGAGCTGCCTGGGGCCAGGGGCAGATGCTGCCACCTCAGGGCCAGACCCACAGAGGCAGCGGGGGAGGAAGGGTGGTCTGCCTCTCTGGCCTGCGGTTGGGGAATGGGGTGTGGGAAGGCAGGAACAGAGGGCTTCCTGGGCTCCTGAGTCCAGGACCTGTGGGGTCAGCTTGGGAGCTCAGCTGAGGCGCTGGCCTCAGGCAC

**Gln54Arg**

**818A>G**

ATGCTCATTCCCCCACTCACACGGCCTCCAGATGCGCGTGCTGCAGGCGGTCCTGCCGCCCCTGCCTCAGGTGGTGTGCACCTACCGTGATGTGCGCTTCGAGTCCATCCGGCTCCCTGGCTGCCCGCGTGGTGTGGACCCCGTGGTCTCCTT

##### Gly102Ser

**961G>A**

CCCTGTGGCTCTCAGCTGTCGCTGTGGACCCTGCCGCCGCAGCACCTCTGACTGTGGGGGTCCCAAAGACCACCCCTTGACCTGTGACCACCCCCAACTCTCAGGCCTCCTCTTCCTCTAAAGACCCTCCCCGCAGCCTTCCAAGTCCATCCCGACTCCTGGAGCCCTGACACCCCGATCCTCCCACAATAAAGGCTTCTCAATCCGCA

## Homo sapiens chorionic gonadotropin, beta polypeptide (*CGB*), 19q13.32

GenBank Reference sequence NC_000019.8; GI:42406306. Chromosome region from 54217939 to 54219405 base; 1467 bp

TCCAGCACCTTTCTCGGGTCACGGCCTCCTCCTGGCTCCCAGGACCCCACCATAGGCAGAGGCAGGCCTTCCTACACCCTACTCCCTGTGCCTCCAGCCTCGACTAGTCCCTAGCACTCGACGACTGAGTCTCTGAGGTCACTTCACCGTGGTCTCCGCCTCACCCTTGGCGCTGGACCAGTGAGAGGAGAGGGCTGGGGCGCTCCGCTGAGCCACTCCTGCGCCCCCCTGGCCTTGTCTACCTCTTGCCCCCCGAGGGGTTAGTGTCGAGCTCACCCCAGCATCCTATCACCTCCTGGTGGCCTTGCCGCCCCCACAACCCCGAGGTATAAAGCCAGGTACACGAGGCAGGGGACGCACCAAGGATGGAGATGTTCCAGGTAAGACTGCAGGGCCCCTGGGCACCTTCCACCTCCTTCCAGGCAATCACTGGCATGAGAAGGGGTCAGACCAGTGTGAGCTGTGGAAGGAGGCCTCTTTCTGGAGGAGCGTGACCCCCAGTAAGCTTCAGGTGGGGCATTTCCTGAAGGTGCGGATCTGAAATGTTGGGGTATCTCAGGTCCTCTGGGCTGTGGGGTGGGCTCTGAAAGGCAGGTGTCCGGGTGGTGGGTCCTGAATAGGAGATGCCGGGAAGGGTCTCTGGGTCTTTGTGGGTGGTGTACCACGCGGGATGGGAAGGCCAGGACTCGGGGCTGCGGTCTCAGACCCGGGTGAAGCAGTGTCCTTGTCCCAGGGGCTGCTGCTGTTGCTGCTGCTGAGCATGGGCGGGACATGGGCATCCAAGGAGCCGCTTCGGCCACGGTGCCGCCCCATCAATGCCACCCTGGCTGTGGAGAAGGAGGGCTGCCCCGTGTGCATCACCGTCAACACCACCATCTGTGCCGGCTACTGCCCCACCATGGTGAGCTGCCCGGGGCCGGGGCAGGTGCTGCCACCTCAGGGCCAGACCCACAGAGGCAGCGGGGGAGGAAGGGTGGTCTGCCTCTCTGGTCAGGGGCTGCGGAATGGGGTGTGGGAGGGCAGGAACAGAGGGCTTCCTGGACCCCTGAGTCTGAGACCTGTGGGGGCAGCTGGGGAGCTCAGCTGAGGCGCTGGCCCCAGGCACATGCTCATTCCCCCACTCACACGGCTTCCAGACCCGCGTGCTGCAGGGGGTCCTGCCGGCCCTGCCTCAGGTGGTGTGCAACTACCGCGATGTGCGCTTCGAGTCCATCCGGCTCCCTGGCTGCCCGCGCGGCGTGAACCCCGTGGTCTCCTACGCCGTGGCTCTCAGCTGTCAATGTGCACTCTGCCGCCGCAGCACCACTGACTGCGGGGGTCCCAAGGACCACCCCTTGACCTGTGATGACCCCCGCTT

**Asp117Ala**

**1363A>C**

CCAGGACTCCTCTTCCTCAAAGGCCCCTCCCCCCAGCCTTCCAAGCCCATCCCGACTCCCGGGGCCCTCGGACACCCCGATCCTCCCACAATAAAGGCTTCTCAATCCGC

## Homo sapiens chorionic gonadotropin, beta polypeptide 5 (*CGB5*), 19q13.32

GenBank Reference sequence NC_000019.8; GI:42406306. Chromosome region from 54238914 to 54240380 base; 1467 bp

TCCAGCACTTTGCTCGGGTCACGGCCTCCTCCTGGCTCCCAGGACCCCACCATAGGCAGAGGCAGGCCTTCCTACACCCTACTCCCTGTGCCTCCAGGCTCGACTAGTCCCTAGCACTCGACGACTGAGTCTCTGAGATCACTTCACCGTGGTCTCCGCCTCACCCTTGGCGCTGGACCAGTGAGAGGAGAGGGCTGGGGCGCTCCGCTGAGCCACTCCTGCGCCCCCCTGGCCTTGTCTACCTCTTGCCCCCCGAAGGGTTAGTGTCGAGCTCACCCCAGCATCCTACAACCTCCTGGTGGCCTTGCCGCCCCCACAACCCCGAGGTATAAAGCCAGGTACACGAGGCAGGGGACGCACCAAGGATGGAGATGTTCCAGGTAAGACTGCAGGGCCCCTGGGCACCTTCCACCTCCTTCCAGGCAATCACTGGCATGAGAAGGGGCAGACCAGTGTGAGCTGTGGAAGGAGGCCTCTTTCTGGAGGAGCGTGACCCCCAGTAAGCTTCAGGTGGGGCAGTTCCTAAGGGTGGGGATCTGAAATTTTGGGGCATCTCAGGTCCTCTGGGCTGTGGGGTGGGCTCTGAAAGGCAGGTGTCCGGGTGGTGGGTCCTGAATAGGAGATGCCGGGAAGGGTCTCTGGGTCTTTGTGGGTGGTGTACCACGCGGGATGGGAAGGCCAGGACTCGGGGCTGCGGTCTCAGACCCGGGTGAAGCAGTGTCCTTGTCCCAGGGGCTGCTGCTGTTGCTGCTGCT

**Arg6Gln**

**794G>A**

GAGCATGGGCGGGACATGGGCATCCAAGGAGCCGCTTCGGCCACGGTGCCGCCCCATCAATGCCACCCTGGCTGTGGAGAAGGAGGGCTGCCCCGTGTGCATCACCGTCAACACCACCATCTGTGCCGGCTACTGCCCCACCATGGTGAGCTGCCCGGGGCCCGGGCAGGTGCTGCCACCTCAGGGCCAGACCCACAGAGGCAGCGGGGGAGGAAGGGTGGTCT

**1038C>T** GCCTCTCTGGTCAGGGGCTGCGGAATGGGGTGTGGGAGGGCAGGAACAGAGGGCTTCCCGGACCCCTGAGTCTGAGACCTGTGGGGGCAACTGGGGAGCTCAGCTGAGGCGCTGGCCCCAGGCACATGCTCATTCCCCCACTCACACGGC

**Val56Leu**

**1178G>C**

TTCCAGACCCGCGTGCTGCAGGGGGTCCTGCCGGCCCTGCCTCAGGTGGTGTGCAACTACCGCGATGTGCGCTTCG

**Val79Met**

**1247G>A**

AGTCCATCCGGCTCCCTGGCTGCCCGCGCGGCGTGAACCCCGTGGTCTCCTACGCCGTGGCTCTCAGCTGTCAATGTGCACTCTGCCGCCGCAGCACCACTGACTGCGGGGGTCCCAAGGACCACCCCTTGACCTGTGATGACCCCCGCTTC

**Asp117Ala**

**1362A>C**

CAGGACTCCTCTTCCTCAAAGGCCCCTCCCCCCAGCCTTCCAAGTCCATCCCGACTCCCGGGGCCCTCGGACACCCCGATCCTCCCACAATAAAGGCTTCTCAATCCGCA

## Homo sapiens chorionic gonadotropin, beta polypeptide 7 (*CGB7*), 19q13.32

GenBank Reference sequence NC_000019.8; GI:42406306. Chromosome region from 54249343 to 54250809 base; 1467 bp

TCCAGCACCTTTCTCGGGTCACGGCCTCCTCCTGGTTCCCAAGACCCCACCATAGGCAGAGGCAGGCCTTCCTACACCCTACTCTCTGTGCCTCCAGCCTCGACTAGTCCCTAGCACTCGACGACTGAGTCTCAGAGGTCACTTCACCGTGGTCTCCGCCTCATCCTTGGCGCTAGACCACTGAGGGGAGAGGACTGGGGTGCTCCGCTGAGCCACTCCTGTGCCTCCCTGGCCTTGTCTACTTCTCGCCCCCCGAAGGGTTAGTGTCCAGCTCACTCCAGCATCCTACAACCTCCTGGTGGCCTTGACGCCCCCACAAACCCGAGGTATAAAGCCAGGTACACCAGGCAGGGGACGCACCAAGGATGGAGATGTTCCAGGTAAGACTGCAGGGCCCCTGGGCACCTTCCACCTCCTTCCAGGCCATCACTGGCATGAGAAGGGGCAGACCAGTGTGAGCTGTGGAAGGACGCCTCTTTCTGGAGGAGCGTGACCCCCAATAAGCTTCACGTGGGGCAGTTCCTGAGGGTGGGGATCTGAAATGTTGGGGCATCTCAGGTCCCTCGGGCTGTGGGGTGGGCTCTGAAAGGCAGGTGTCCGGGTGGTGGGTCCTGAATAGGAGATGCCGGGAAGGGTCTCTGGGTCTTTGTGGGTGGTGTACCCTGGGGGATGGGAAGGCCAGGGCTCAGGGCTGTGGTCTCAGGCCCGGGTGAAGCAGTGTCCTTGTCCCAGGGGCTGCTGCTGTTGCTGCTGCTG

Arg2Lys Met4Pro

#### 782G>A 787/788AT>CC

AGCATGGGCGGGACATGGGCATCCAGGGAGATGCTTCGGCCACGGTGCCGCCCCATCAATGCCACCCTGGCTGTGGAGAAGGAGGGCTGCCCCGTGTGCATCACCGTCAACACCACCATCTGTGCCGGCTACTGCCCCACCATGGTGAGCTGCCCGGGGCCCGGGCAGGTGCTGCCACCTCAGGGCCAGACCCACAGAGGCAGCGGGGGAGGAAGGGTGGTCTGCCTCTCTGGTCAGGGGCTGCGGAATGGGGTGTGGGAGGGCAGGAACAGAGGGCTTCCTGGACCCCTGAGTCTGAGACCTGTGGGGGCAGCTGGGGAGCTCAGCTGAGGCGCTGGCCCCAGGCACATGCTCATTCTCCCACTCACACGGCTT

Ala51Thr

1162G>A

CCAGACCCGCGTGCTGCAGGGGGTCCTGCCGGCCCTGCCTCAGGTGGTGTGCAACTACCGCGATGTGCGCTTCGA

#### Arg74Cys

#### 1232C>T

GTCCATCCGGCTCCCTGGCTGCCCGCGCGGCGTGAACCCCGTGGTCTCCTACGCCGTGGCTCTCAGCTGTCAATGTGCACTCTGCCGCCGCAGCACCACTGACTGCGGGGGTCCCAAGGACCACCCCTTGACCTGTGATGACCCCCGCTTCC

##### Ala117Asp

##### 1363C>A

AGGCCTCCTCTTCCTCAAAGGCCCCTCCCCCCAGCCTTCCAAGTCCATCCCGACTCCCGGGGCCCTCAGACACCCCGATCCTCCCACAATAAAGGCTTCTCAATCCGCA

## Homo sapiens chorionic gonadotropin, beta polypeptide 8 (*CGB8*), 19q13.32

GenBank Reference sequence NC_000019.8; GI:42406306. Chromosome region from 54242707 to 54244180 base; 1474 bp

GCTTCAGTCCAGCACCTTTCTCGGGTCACGGCCTCCTCCTGGCTCCCAGGACCCCACCATAGGCAGAGGCAGGCCTTCCTACACCCTACTCCCTGTGCCTCCAGGCTCGACTAGTCCCTAGCACTCGACGACTGAGTCTCTGAGGTCACTTCACCGTGGTCTCCGCCTCACCCTTGGCGCTGGACCAGTGAGAGGAGAGGGCTGGGGCGCTCCGCTGAGCCACTCCTGCGCCCCCCTGGCCTTGTCTACCTCTTGCCCCCCGAAGGGTTAGTGTCGAGCTCACTCCAGCATCCTACAACCTCCTGGTGGCCTTGCCGCCCCCACAACCCCGAGGTTTAAAGCCAGGTACACGAGGCAGGGGACACACCAAGGATGGAGATGTTCCAGGTAAGACTGCAGGGCCCCTGGGCACCTTCCACCTCCTTCCAGGCAATCACTGGCATGAGAAGGGGCAGACCAGTGTGAGCTGTGGAAGGAGGCCTCTTTCTGGAGGAGCGTGACCCCCAGTAAGCTTCAGGTGGGGCATTTCCTGAAGGTGGGGATCTGAAATGTTGGGGTATCTCAGGTCCTCTGGGCTGTGGGGTGGGCTCTGAAAGGCAGGTGTCCGGGTGGTGGGTCCTGAATAGGAGATGCCGGGAAGGGTCTCTGGGTCTTTGTGGGTGGTGTACCACGTGGGATGGGAAGGCCAGGACTCGGGGCTGCGGTCTCAGACCCGGGTGAAGCAGTGTCCTTGTCCCAGGGGCTGCTGCTGTTG

**Arg8Trp**

**806C>T**

CTGCTGCTGAGCATGGGCGGGACATGGGCATCCAAGGAGCCGCTTCGGCCACGGTGCCGCCCCATCAATGCCACC

**Val29Ile**

**869G>A**

CTGGCTGTGGAGAAGGAGGGCTGCCCCGTGTGCATCACCGTCAACACCACCATCTGTGCCGGCTACTGCCCCACCATGGTGAGCTGCCCGGGGCCGGGGCAGGTGCTGCCACCTCAGGGCCAGACCCACAGAGGCAGCGGGGGAGGAAG

**1045C>T**

GGTGGTCTGCCTCTCTGGTCAGGGGCTGCGGAATGGGGTGTGGGAGGGCAGGAACAGAGGGCTTCCCGGACCCCTGAGTCTGAGACCTGTGGGGGCAGCTGGGGAGCTCAGCTGAGGCGCTGGCCCCAGGCACATGCTCATTCTCCCACTCACACGGCTTCCAGACCCGCGTGCTGCAGGGGGTCCTGCCGGCCCTGCCTCAGGTGGTGTGCAACTACCGCGATGT

**Pro73Arg**

**1237C>G**

GCGCTTCGAGTCCATCCGGCTCCCTGGCTGCCCGCGCGGCGTGAACCCCGTGGTCTCCTACGCCGTGGCTCTCAGCTGTCAATGTGCACTCTGCCGCCGCAGCACCACTGACTGCGGGGGTCCCAAGGACCACCCCTTGACCTGTGATGACCCCCGCTTCCAGGACTCCTCTTCCTCAAAGGCCCCTCCCCCCAGCCTTCCAAGTCCATCCCGACTCCCGGGGCCCTCGGACACCCCGATCCTCCCACAATAAAGGCTTCTCAATCCGCA

**Figure S2.** Aminoacid sequence alignment for the products of HCG beta-subunit coding genes (*CGB, CGB5, CGB7, CGB8*), *LHB*, and *CGB1* and *CGB2*.

CGB5 1 -----MEMFQGLLLLLLLSMGGTWASKEPLRPRCRPINATLAVEKEGC-PVCITVNTTIC

CGB8 1 -----...........................................-...........

CGB 1 -----...........................................-...........

CGB7 1 -----.....................R.M...................-...........

LHB 1 -----...L.............A...R.....W.H....I........-...........

CGB1 1 MSTFPVLAEDIP.RERHVKEAAAV.AA.HG.DMGIQGA.SAT.PPHQ.H.G.GEG--GLP

CGB2 1 MSTSPVLAEDIP.RERHVKGAAAV.AA.HG.DMGIQGA.SAT.PPHQ.H.G.GEG--GLP

CGB5 55 AGYCPTMTRVLQGVLPALPQVVCNYRDVRFESIRLPGCPRGVNPVVSYAVALSCQCALCR

CGB8 55 ............................................................

CGB 55 ............................................................

CGB7 55 ............................................................

LHB 55 .......M....A...P......T..................D....FP.....R.GP..

CGB1 59 RVHHRQHHHLCRLLPHHD.RAAGGPAGPASGGVQ..R.ALR.H.APWLPARREPRGL.R.

CGB2 59 RVHHRQHHHLCRLLPHHD.RAAGGPAGPASGGVQ..R.ALR.H.APWLPARREPRGL.R.

CGB5 115 RS-TTDCGGPKDHPLTCDDPRFQDSSSSKAPPPSLPSPSRLPGPSDTPILPQ

CGB8 115 ..-.................................................

CGB 115 ..-.................................................

CGB7 115 ..-....................A............................

LHB 115 ..-.S.............H.QLSGLLFL------------------------

CGB1 119 G.QLSM.TL.PQ.H--------------------------------------

CGB2 119 G.QLSM.TL.PQ.H--------------------------------------
